# Supplementary material for: Apolipoprotein E (ApoE) Rescues the Contractile Smooth Muscle Cell Phenotype in Popliteal Artery Aneurysm Disease
Source: Biomolecules. 2023 Jul 4;13(7):1074. doi: 10.3390/biom13071074 (PMC10377618; doi:10.3390/biom13071074)
Supplement: Supplementary file 1 [file biomolecules-13-01074-s001.zip › Supplement Material.pdf]

Data Supplement

Supplementary Figures and Figure Legends

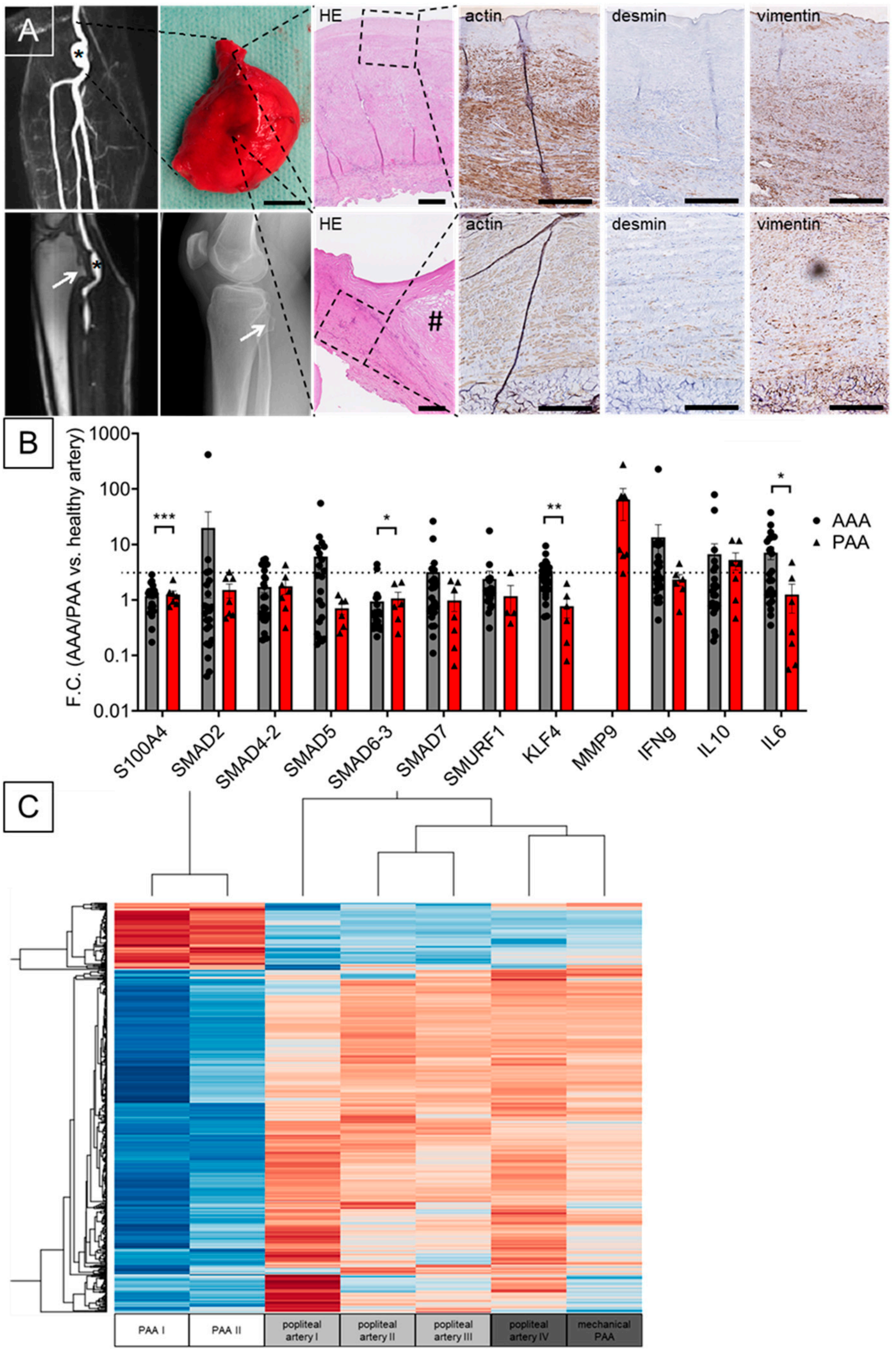

Suppl. Figure S1: False aneurysm case presentation, histologic analysis, qPCR results from comparative analysis and gene expression heatmap. (A) Possible mechanically induced PAA (asterisk) by osseous hypertrophy (arrow) shows a different morphology with a bulla-like structure (#). VSMC marker desmin is not lost and is similarly expressed in the aneurysmatic and non-aneurysmatic portion (scale bar 100µm). **(B)** Gene Expression Analysis of PAA and AAA shown as fold change vs. the respective non-aneurysmatic vessel (AAA vs. aorta: n= 24 vs. n=10; PAA vs. popliteal artery: n= 7 vs. n=8; PAA vs. AAA: unpaired t-test, * p<0.05; MMP only available from AAA patients). **(C)** Gene expression heatmap with hierarchical clustering for PAA vs. popliteal artery. Of note, the false aneurysm PAA shows a similar gene expression pattern as non-dilated popliteal arteries (popliteal artery IV and mechanical PAA from the reported patient).

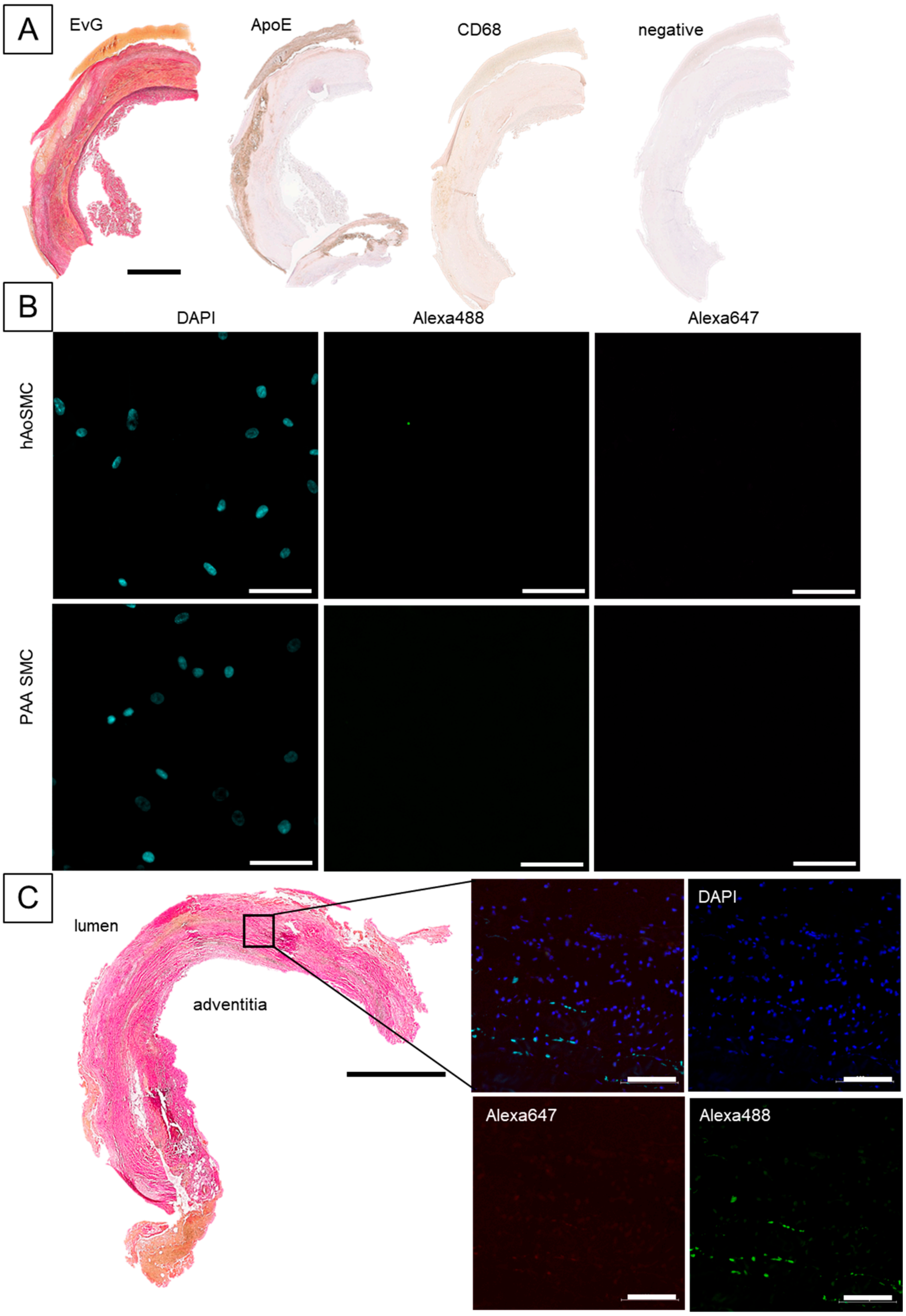

Suppl. Figure S2: PAA immunohistochemistry and fluorescence imaging negative controls. (A) ApoE is also expressed close to or by CD68 positive cells in PAA tissue samples (scale bar 2mm). **(B)** Appropriate negative controls of primary human cell culture stainings (scale bar 100µm). **(C) Appropriate Negative Control to** PAA tissue immunofluorescence(scale bar 100 µm, (HE; scale bar 2 mm)

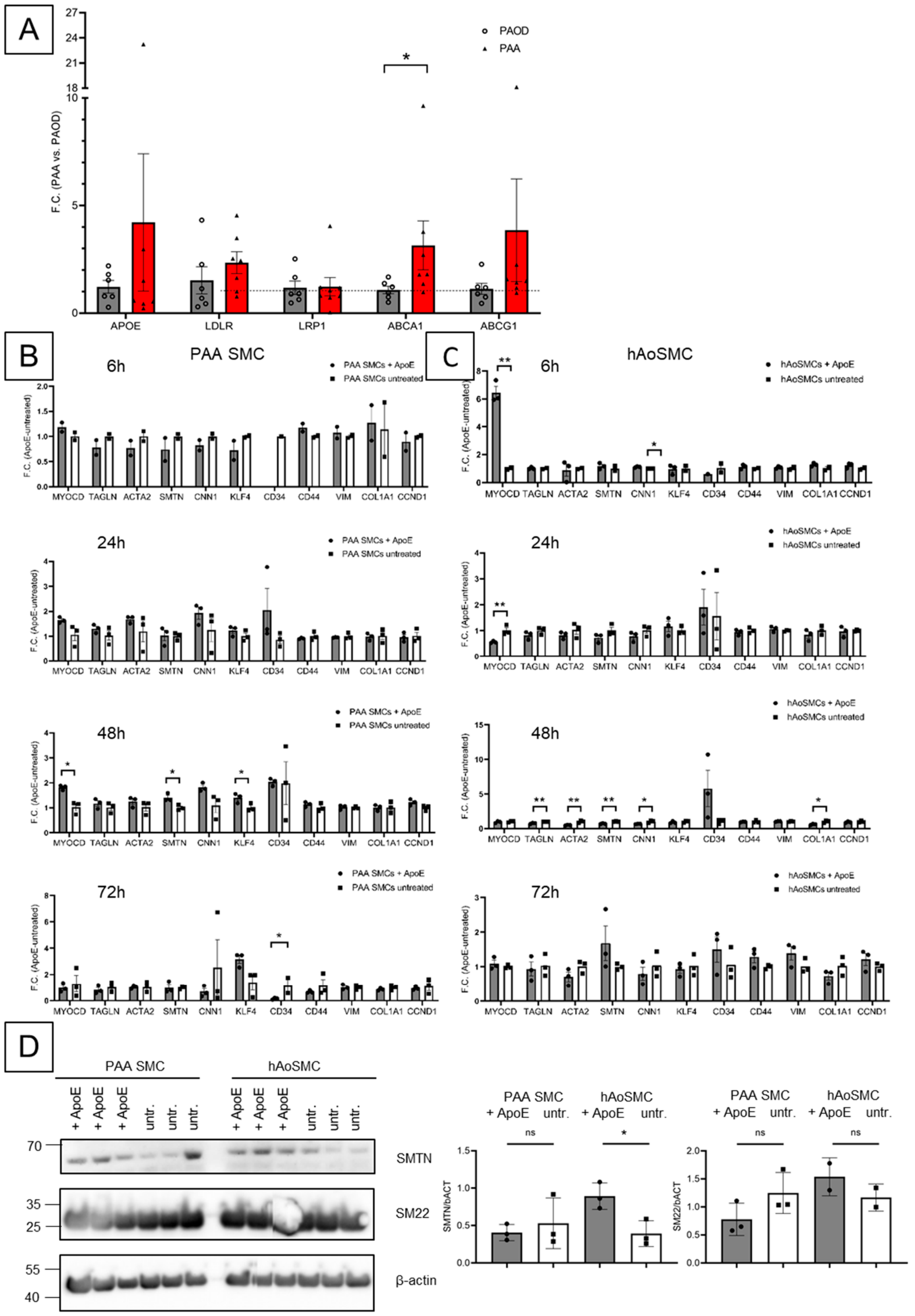

Suppl. Figure S3: Cholesterol hemostasis gene panel and primary cell gene expression results. (A) Gene expression analysis of *APOE*, low-density lipoprotein receptor-related protein 1 (*LRP1*), phospholipid-transporting ATPase (*ABCA1*), and ATP-binding cassette dub-family G Member 1 (*ABCG1*) in PAA (n=7) vs. PAOD (n=6) tissue. **(C)** Contractile VSMC markers gene expression after 6h, 24h, 48h, and 72h APOE treatment in PAA SMCs and hAoSMCs (both 50µg/ml ApoE) (unpaired t-test, * p<0.05, ** p<0.01, *** p<0.001, Mean +/- SEM). **(D)** WB and quantification for SMTN and SM22 normalized to ß-actin for PAA SMCs and hAoSMCs upon ApoE stimulation (* = p<0.05; ns= not significant).

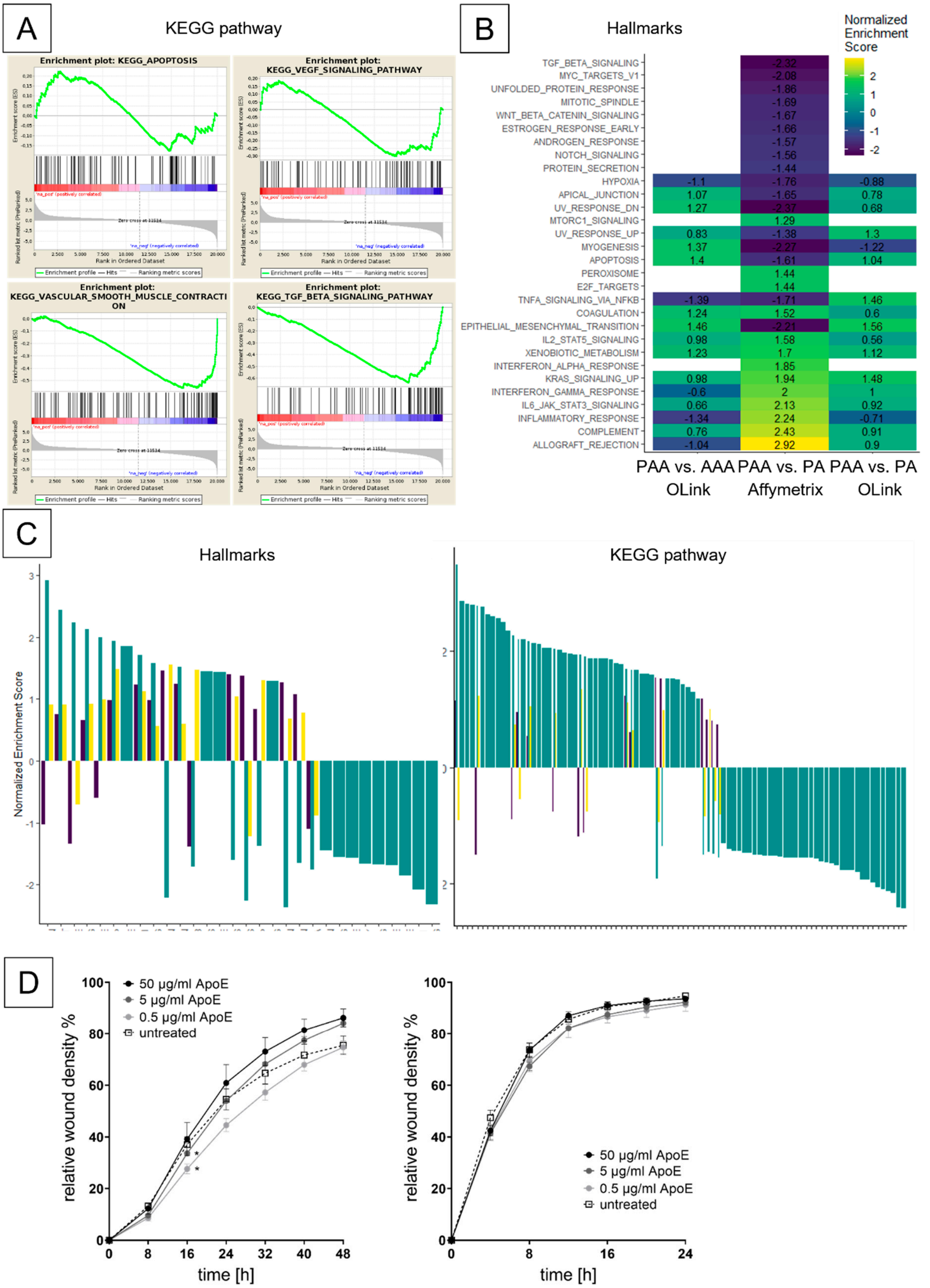

Suppl. Figure S4: Comparative pathway enrichment analysis. (A) Selected KEGG pathway enrichment plots. **(B)** Comparative Hallmark enrichment analysis for gene expression (Affymetrix) and protein expression (OLink) data based on the highest (negative/positive) enrichment score from gene expression from PAA vs. popliteal artery (PA). **(C)** Similar depiction comparing Hallmark and KEGG enrichment. **(D)** Live cell imaging migration assay depicts no changes in relative wound density over time with different ApoE treatment

concentrations for primary PAA patient-derived cells (left) and human aortic smooth muscle cells (right) (two-way ANOVA treated vs. untreated, * p<0.05, ** p<0.01, *** p<0.001, **** p<0.0001; Mean +/- SEM).

Full Membrane – CNN1 (upper) and ß-actin (lower)

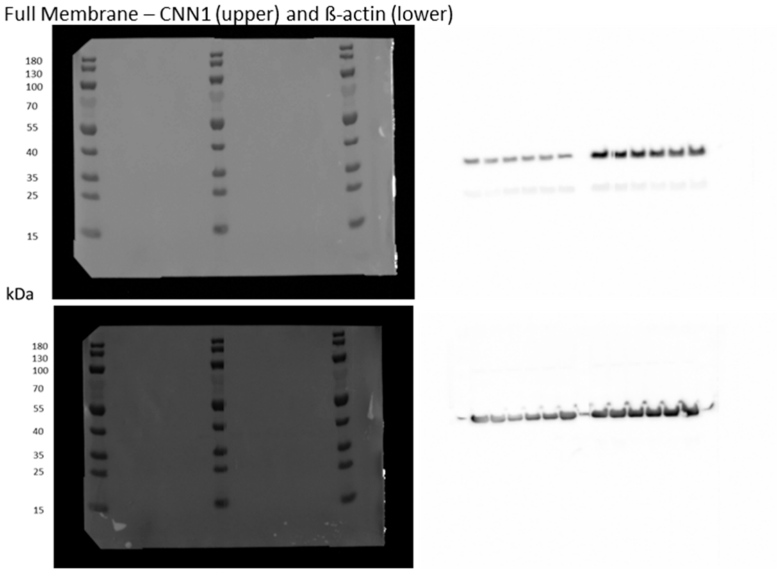

Full Membrane –TAGLN/SM22 (upper) and ß-actin (lower)

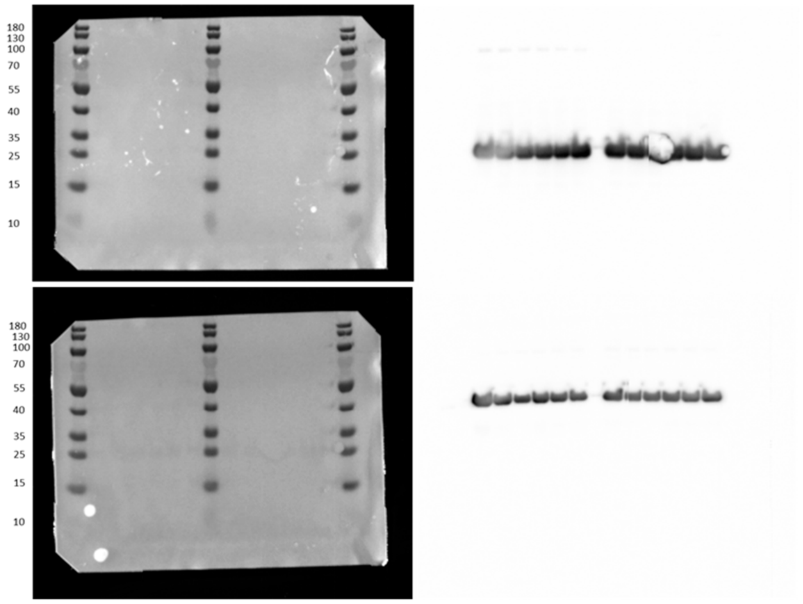

Full Membrane –SMTN (upper) and ß-actin (lower

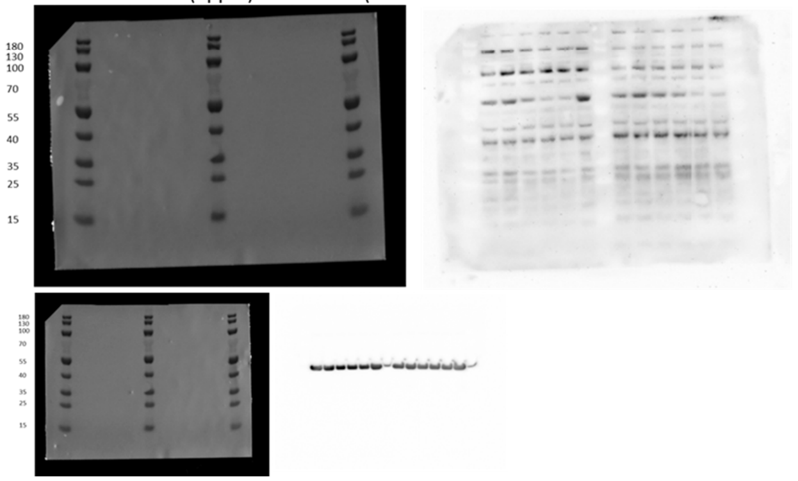

Suppl. Figure S5: WB full membranes. The full membranes are shown for all three proteins investigated, each in combination with the respective ß-actin loading control staining.

Supplement Tables

	**N=16 PAA**<br>**N=8 popliteal arteries**<br>from<br>**N=14 patients**	**N=6 PAOD**	**N=24 AAA**	**N=10 aorta**
**age** (y)	68.5 ± 8.7	64.3 ± 12.4	67.3 ± 9.1	62.2 ± 13.1
**sex** (f/m)	0/12	0/6	2/22	3/7
**vessel diameter** (mm)	29 ± 4 (norm <12)	n.a.	58 ± 9.2	n.a.
**bilateral PAA**	66,7%	n.a.	n.a.	n.a.
**other aneurysm**	33.3% AAA	n.a.	8.3% PAA	n.a.
**leukocyte count** ($*10^3/\mu l$)	<9 (norm <9)	-	10.2 ± 3.4	<9
**comorbidities**				
**- hypertensive disease**	66.7%	60%	85%	90%
**- NIDDM**	25%	16.7%	12.5%	0
**- CAD**	16.7%	33.3%	45.8%	20%
**- stroke**	33.3%	-	8.3%	0
**- smoking**	83%	83.3%	95.8%	100%
**- COPD**	16.7%	0	12.5%	20%

Suppl. Table S1: Detailed patients' characteristics. The table shows the patient data corresponding to the vascular tissue samples. Mean, and standard deviation are shown where applicable and normal values are included in parentheses. Smoking includes current and past tobacco abuse. Eight PAA samples had matched control arteries from the same individual's non-dilated part of the respective artery. (CRP: C-reactive protein; NIDDM: non-insulin-dependent diabetes mellitus; CAD: coronary artery disease; COPD: chronic obstructive pulmonary disease)

Gene (ID)	Name	regulation in PAA vs. PA
CNN1 (1264)	calponin 1, basic, smooth muscle	down
MYH11 (4629)	myosin, heavy chain 11	down
PLN (5350)	*phospholamban*	down
SORBS1 (10580)	*sorbin and SH3 domain containing 1*	down
ITGA8 (8516)	*integrin, alpha 8*	down
NR4A1 (3164)	*nuclear receptor subfamily 4A1*	down
PPP1R14A (94274)	*protein phosphatase 1*	down
SYNPO2 (171024)	*synaptopodin 2*	down
FHL1 (2273)	four and a half LIM domains 1	down
SYNM (23336)	synemin, intermediate filament protein	down
PPP1R12B (4660)	*protein phosphatase 1*	down
MMP9	matrix metalloproteinase 9	up
MMP7	matrix metalloproteinase 7	up
APOC1 (341)	apolipoprotein C-I	up
SPP1 (6696)	secreted phosphoprotein 1	up
APOE (348)	apolipoprotein E	up
IFI30//PIK3R2 (10437)	*interferon, gamma-inducible protein 30*	up

Suppl. Table S2: Annotations of the significant genes in PAA vs. popliteal artery gene expression analysis from **Fig. 2A**. All genes previously identified of relevance in AAA or VSMC phenotype switch (and APOE/C1) size 11;

Suppl. Table S3: Excel file with Affymetrix results; The table is sorted by p-value and lists the gene symbol, gene name, and Affymetrix-ID along with the expression values.

Suppl. Table S4: Excel file with OLink results.
